# Public human microbiome data are dominated by highly developed countries

**DOI:** 10.1371/journal.pbio.3001536

**Published:** 2022-02-15

**Authors:** Richard J. Abdill, Elizabeth M. Adamowicz, Ran Blekhman

**Affiliations:** 1 Department of Genetics, Cell Biology, and Development, University of Minnesota, Minneapolis, Minnesota, United States of America; 2 Department of Ecology, Evolution and Behavior, University of Minnesota, St. Paul, Minnesota, United States of America; New York University School of Medicine, UNITED STATES

## Abstract

The importance of sampling from globally representative populations has been well established in human genomics. In human microbiome research, however, we lack a full understanding of the global distribution of sampling in research studies. This information is crucial to better understand global patterns of microbiome-associated diseases and to extend the health benefits of this research to all populations. Here, we analyze the country of origin of all 444,829 human microbiome samples that are available from the world’s 3 largest genomic data repositories, including the Sequence Read Archive (SRA). The samples are from 2,592 studies of 19 body sites, including 220,017 samples of the gut microbiome. We show that more than 71% of samples with a known origin come from Europe, the United States, and Canada, including 46.8% from the US alone, despite the country representing only 4.3% of the global population. We also find that central and southern Asia is the most underrepresented region: Countries such as India, Pakistan, and Bangladesh account for more than a quarter of the world population but make up only 1.8% of human microbiome samples. These results demonstrate a critical need to ensure more global representation of participants in microbiome studies.

## Abstract 2

The importance of sampling from globally representative populations has been well established in human genomics, but what about the microbiome? This study shows that metadata from almost half a million samples reveals worldwide human microbiome research is skewed heavily in favor of Europe and North America and excludes large but less developed nations in Asia and Africa.

## Background

A growing body of research shows that the human microbiome has broad relevance to human health and disease. However, identifying the specific connections between the microbiome and human health requires a broad survey of both human populations and their most common health conditions. Even among healthy individuals, human microbiome composition varies between populations in ways that are still being uncovered: Geography and geographic relocation has been found to have an influence on microbiome composition [1–3], as have host genetic variation and ethnicity [4–6]. Diet [7], lifestyle [8], and patterns in antibiotic use [9] have all been linked to microbiome composition, with other studies considering the influence of locational factors such as pollution [10]. Even within countries, interacting factors such as income, race, and education have critical impacts on health outcomes that could be mediated by the human microbiome [11]. Some microbiome studies have specifically collected and compared data from global sites [12,13], but large gaps and disparities still exist in which microbiomes are being studied on a global scale. The human microbiome has been linked to a growing number of social, medical, and economic factors not directly related to host genetics, which reinforces the urgent need to evaluate the microbiomes of many populations [11,14].

Other genomics fields have developed similar gaps, in which disproportionate attention is paid to the majority populations of wealthy countries: Genome-wide association studies (GWASs), for example, have been primarily conducted in populations with European ancestry [15,16]. As a result, polygenic risk scores (PRSs) from these studies have poorer accuracy when applied to non-European groups, limiting the possible benefits of this research—including personalized medicine, early disease screening, and risk prediction—to European-descended populations [17–19]. There has been a concerted effort in genomics to include non-European individuals in GWAS studies, concurrent with calls to build research infrastructure and capacity globally [16]. It is likewise critical to identify underrepresented populations and locations in both genomics and microbiome research; otherwise, the benefits of host–microbiome research may only extend to a subset of the global population.

To investigate the geographic distribution of microbiome studies, we used metadata on all human microbiome datasets in the BioSample database, which includes metadata describing samples in the Sequence Read Archive (SRA), DNA Data Bank of Japan, and European Nucleotide Archive [20]. Our data include the country of origin and time of release for more than 444,000 samples, including both 16S amplicon sequencing and shotgun metagenomic sequencing, released over the last 11 years. These samples from the 3 largest genomic databases represent a large majority of all human microbiome samples that have been published.

## Results

We downloaded metadata for 444,829 human microbiome samples across 19 body sites and 2,592 studies. These data are available from the BioSample database maintained by the National Center for Biotechnology Information (NCBI), which includes metadata describing raw sequencing data deposited in multiple international repositories, including SRA [21]. While sample-level genomic sequencing data are uploaded to SRA, information such as geographic origin is saved separately to an entry in the BioSample database. BioSamples can be tagged with any number of “attributes,” including 485 standardized fields documented by NCBI [22]; we downloaded all attributes for all these samples. We used a Python script to load this metadata into a PostgreSQL database, where the information was aggregated using sample metadata such as country of origin and time of publication (see **Materials and methods**).

**Fig 1:**
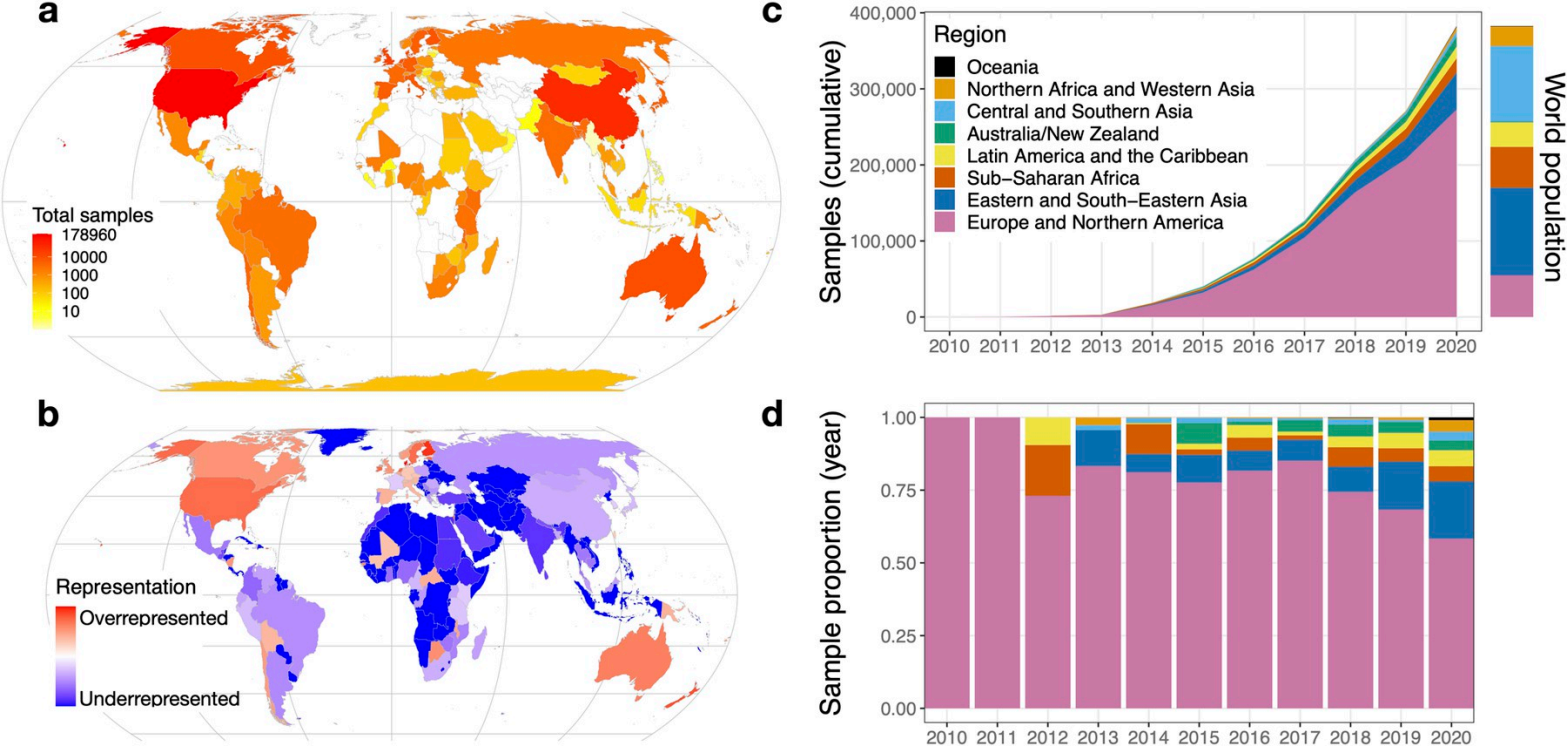
Global microbiome representation. **(a)** Total samples by country. The color of each country indicates the total number of samples originating in that country using a log10 scale. **(b)** Relative representation by country. The color of each country indicates its representation in human microbiome datasets, relative to its share of world population. Red colors mark countries that are overrepresented relative to their population, and blue colors mark countries that are underrepresented. Countries with zero samples in the dataset are marked with dark blue. **(c)** Cumulative microbiome samples by world region. The x-axis indicates the year, and the y-axis indicates the cumulative microbiome samples available at the end of that year. Colors indicate the cumulative microbiome samples from each of the world regions specified in the legend. The colored bar to the right of the plot indicates the share of the world population living in each of the regions using the same colors. **(d)** Proportion of annual samples. The x-axis indicates the year, and the y-axis indicates the proportion of samples from each world region published in that year. Colors correspond to the world regions shown in panel C. The data and code needed to generate this figure can be found at https://doi.org/10.5281/zenodo.5351179. All maps are based on public domain Natural Earth data; the base layer is available for download at https://www.naturalearthdata.com/http//www.naturalearthdata.com/download/50m/cultural/ne_50m_admin_0_countries.zip.

As expected, we found that the number of human microbiome samples with publicly available data has been increasing over time, from 3 microbiome samples in 2010 to 123,302 in 2020, the first year in which more than 100,000 human microbiome samples were released (**S1 Fig**). Although there were microbiome studies conducted prior to 2010, that was the first year of the BioSample database, which all depositors must now use if they submit sequencing data to the SRA. The most commonly used attribute in this subset of samples is the geographic origin of the sample [22], which is available for 99.5% of samples (**S1 Table**). Using this attribute, we were able to determine the country of origin for 382,711 (86%) human microbiome samples (**Fig 1A**), which originated in 115 different countries. We found that 178,960 samples (40.2%) were from the US, almost 5 times more than any other country (**Table 1**). China has the next most samples, with 36,162 (8.1%), followed by the United Kingdom, Denmark, Australia, and the Netherlands. China is the only Asian country in the top 14; the first South American country is Chile, in 16th place with 3,616 samples (0.8%). Malawi is the first African country, in 19th place with 3,052 samples (0.7%).

**Table 1:** Samples per country.

Position	Country	Samples	Share
1	US	178,960	40.2%
	Unknown	62,118	14.0%
2	China	36,162	8.1%
3	UK	16,076	3.6%
4	Denmark	11,497	2.6%
5	Australia	9,266	2.1%
6	the Netherlands	9,173	2.1%
7	Canada	8,829	2.0%
8	Finland	7,855	1.8%
9	Italy	6,265	1.4%
10	Germany	5,531	1.2%
11	Spain	5,517	1.2%
12	Sweden	5,248	1.2%
13	Israel	4,831	1.1%
14	New Zealand	4,354	1.0%
15	Japan	4,298	1.0%
16	Chile	3,616	0.8%
17	Bangladesh	3,502	0.8%
18	France	3,402	0.8%
19	Malawi	3,052	0.7%
20	India	2,997	0.7%
	Rest of world	52,280	11.8%

We also evaluated patterns specific to body sites. The number of countries represented in each body site is roughly proportional to the number of overall samples, with the most frequently sampled body site, the human gut, also holding data from the most countries, 96 (**Table 2**). This number drops quickly, however: For example, there are 44 countries represented in the skin microbiome category, and only 22 in the nasopharyngeal microbiome. Even if we consider only the 115 countries that appear in this dataset, it appears most body sites exclude most countries. When we consider body sites per country, rather than countries per body site, we can also evaluate the best characterized country-level microbiomes: China has samples in 17 of the 19 body sites, the most of any country (**S3 Table**), followed by the US with 16. The first South American country on the list, Brazil, has only 9, and South Africa, the first African country, appears in 8 body sites. Next, we used these data to assess country-level patterns at the 5 most prevalent body sites: the gut, mouth, skin, vagina, and lung (**S2 Table**). The US has the most samples in all 5. The skin microbiome category differs notably from the overall top 10: Although the US and China again appear at the top, the remainder of the top 10 includes Chile, Bangladesh, Papua New Guinea, Hong Kong, India, Puerto Rico, Australia, and Peru. However, this is also the body site with the most lopsided difference between the US and the rest of the world: The US total (19,706 samples) is 12.6 times that of the number 2 country, China (1,562 samples), and more than 50 times that of the 10th country, Peru (391 samples).

**Table 2:** Samples by body site. Each row indicates a body site related to the human microbiome. The “Samples” column indicates the total number of samples categorized under each body site, and the “Countries” column indicates the number of unique countries with at least 1 sample in that category. Samples without a known country are included in the sample count, but not the country count. Body sites map directly to categories defined in the NCBI Taxonomy Browser; see **S5 Table** for a list of category IDs combined for each body site.

Body site	Samples	Countries
Gut	220,017	96
Human metagenome*	69,697	58
Oral	47,798	63
Skin	36,593	44
Vaginal	17,784	31
Lung	17,307	30
Nasopharyngeal	15,646	22
Feces	6,858	13
Reproductive system	3,180	6
Blood	2,707	9
Saliva	2,503	15
Milk	2,060	9
Urinary tract	1,187	4
Tracheal	520	2
Sputum	364	3
Eye	359	8
Semen	203	3
Bile	45	2
Skeleton	1	1

*Samples under the “human metagenome” label refer to an NCBI category that does not specify a particular body site.

NCBI, National Center for Biotechnology Information.

To examine patterns of under- and overrepresentation of countries, we compared human microbiome sample counts to each country’s population, according to United Nations estimates for 2020 [23]. The US is dramatically overrepresented relative to its population: Although the country has about 4.3% of the global population, 40.2% of human microbiome samples originate there. Proportionally, Denmark is the most overrepresented country, with 11,497 samples from a country of about 5.8 million people (**Fig 1B**). Of the 235 countries and territories included in the United Nations population estimates, 120 have zero human microbiome samples available in these public databases.

To gain a better understanding of global representation in microbiome research, we grouped countries using the 8 United Nations Sustainable Development Goals regions [24]. We found that 71.2% of samples with a known location come from Europe and Northern America, a region that holds only 14.3% of the world’s population (**Table 3**). Proportionally, Australia/New Zealand has the most lopsided presence in the database: The region’s 30.3 million people is 0.4% of the population, but accounts for 3.1% of samples (**Fig 1C**). Central/Southern Asia is the most underrepresented region: It holds 25.8% of the population but makes up only 1.8% of microbiome samples. Northern Africa and Western Asia are the next most underrepresented regions, followed by sub-Saharan Africa, which is home to 14.0% of the world’s population but is the source of 4.2% of human microbiome samples. These proportions indicate that a person in Europe or Northern America is roughly 14 times more likely to be studied in a microbiome project than someone from sub-Saharan Africa. The 47 countries on the United Nations list of “least developed countries” account for about 14% of the world’s population [25], but 3.4% of microbiome samples; 29 of those countries have no samples at all (**S4 Table**). We also found that, although samples from Europe and Northern America are overrepresented, in recent years, there is more representation for samples from other regions, most prominently eastern and southeastern Asia (**Fig 1D**).

**Table 3:** Samples and population by region.

Region	Samples	2020 population (estimated, in thousands)	% of samples	% of samples (known location)	% of population	Representation proportion*
Europe and Northern America	272,544	1,116,506	61.3%	71.2%	14.3%	4.97
Eastern and Southeastern Asia	49,007	2,346,709	11.0%	12.8%	30.1%	0.43
Sub-Saharan Africa	18,651	1,094,366	4.2%	4.9%	14.0%	0.35
Latin America and the Caribbean	15,264	653,962	3.4%	4.0%	8.4%	0.49
Australia/New Zealand	13,620	30,322	3.1%	3.6%	0.4%	9.14
Central and Southern Asia	6,685	2,014,709	1.5%	1.7%	25.8%	0.07
Northern Africa and Western Asia	5,621	525,869	1.3%	1.5%	6.7%	0.22
Oceania	1,178	12,356	0.3%	0.3%	0.2%	1.94
Unknown	62,259		14.0%			

Least developed countries	15,254	1,057,438	3.4%	4.0%	13.6%	0.29
Rest of world	367,457	6,737,361	82.6%	96.0%	86.4%	1.11
Unknown	62,118		14.0%			

*Representation proportion calculated by dividing a regions percentage of known samples by its percentage of population.

## Discussion

Our results show that the global distribution of human microbiome sampling is heavily skewed toward North American and European populations, both in total samples (**Fig 1A**) and in samples adjusted for population (**Fig 1B**). The US is by far the greatest contributor to the database (**Table 1**), although this is slowly beginning to change as other countries’ contributions grow (**Fig 1D**). This neglect of most of the world’s population represents a disparity in microbiome research that could limit the health benefits of microbiome research to those countries and populations whose microbiomes have been extensively sampled and studied. Since only a subset of the world’s populations are currently being studied, the associations between the microbiome and disease may not hold in undersampled populations [26,27]. For example, Gupta and colleagues identified several differences in the microbiome of healthy individuals from various geographic locations and lifestyles across the globe; without a consistent “healthy” microbiome across global populations, identifying microbiome–disease associations is nearly impossible [26]. He and colleagues also found that microbiome-based models for predicting metabolic disease failed when applied to populations outside of the geographical location in which they were developed [27]. Additionally, by only sampling a subset of the global population, the diseases studied in the context of the microbiome are limited to diseases which impact that subset. Helminth parasite infections, for example, are common in tropical and subtropical regions of the world, but rare in North American and European populations. Undersampling of the microbiota from populations where these infections are common has led to a lack of clear understanding of the role of the microbiome in helminth colonization and resistance [28].

To ensure greater global equity in the benefits of microbiome research, many stakeholders—funders, researchers, and journals, to name a few—should consider how to ethically prioritize and incentivize improved global representation of microbiome samples, as they have begun to do in genomics with efforts such as the H3Africa initiative [29]. Others have also highlighted opportunities for growth in the microbiome field, such as developing infrastructure and processes in low-resource settings [30,31], building more comprehensive microbial reference databases, and pursuing more flexible and affordable sequencing technologies [32]. Importantly, this approach should be grounded in benefitting the populations and communities sampled, rather than simply using these microbiomes as a tool to improve health in North American and European countries [33,34]. Ongoing discussion of “helicopter research” (e.g., [35]) sheds light on ethical objections to “solving” research disparities with what essentially becomes charity, rather than collaboration: Researchers from wealthy countries obtain funding to do research in developing countries, “helicopter in” to collect data, then leave to publish their papers [36]. The result is more data from that country, but as part of a project that may not address the problems and priorities of the country under study. Local researchers, if they are consulted at all, may be excluded from authorship on the papers that are then hidden behind paywalls, written in a language they may not speak—part of much broader issues in scientific communication [37,38]. Researchers from the so-called “Global North” (as we are) would benefit from deferring to experienced scientists in these countries to find out how to avoid common extractive tropes in imbalanced collaborations (e.g., [35,39]). Research and discussion in other fields may also help scientists trying to build more inclusive research projects: Although there are no easy answers, essays in applied ecology [40–42], ocean science [43], botany [44], geography [45,46], and conservation [47], among many others, deal with the hallmarks and dangers of colonial science [48] and how researchers can change their approach to knowledge production.

The reasons for, and solutions to, global disparities in scientific research go far beyond the scope of this paper, and indeed of the microbiome field. There are broader issues of global representation in science that we and others have discussed, for example, in terms of authorship [49], language [37], and the makeup of editorial boards [40]. The complex history and current conditions driving these disparities requires a comprehensive assessment of global sociopolitical factors that we, as biologists based in North America, are not able to fully address. However, the necessity of such an assessment as a way to solve these problems illustrates an important possible reason that these problems continue to perpetuate. Most microbiome researchers are not trained in social or political science and lack the appropriate tools to assess and address these problems. The more intentional inclusion of social scientists in microbiome projects may help address not only country-level imbalances, but also remediate harmful conventions used to deal with other issues like race [50].

Despite ongoing challenges, there have been several recent success stories of microbiome initiatives set in, driven by, and focused on countries and populations who have been historically left out of microbiome research. One such example is the recently convened Microbiome Task Force from the H3Africa Consortium; their goals are to harmonize and perform meta-analyses of microbiome data from H3Africa, build capacity and knowledge sharing among members, and provide data analysis support to researchers [51]. The Pan-African Bioinformatics Network (H3ABioNet), which has worked extensively in genomics research capacity building in Africa, also recently hosted a hackathon wherein they began work on a data portal for African microbiome samples [52]. In South America, the Brazilian Microbiome project and the recently proposed Ecuadorian Microbiome project both seek to advance microbiome research capacity in their respective countries and create local infrastructure to support these goals [53,54]. Initiatives such as H3Africa’s African Collaborative Center for Microbiome and Genomics Research (ACCME) [55] may be ideally positioned to make progress in these trends, although as research activity grows in these underrepresented countries, using public metadata may become a less viable measure of these disparities: ACCME’s 2 existing microbiome publications, for example, do not have information about data availability [56,57], and ongoing discussions about issues such as data sovereignty [58] raise important questions about whether making data publicly available is a just and sustainable approach to biomedical research in countries or populations with comparatively little power in the global research ecosystem [59–61].

There are several limitations to our study. Metadata quality is the primary hurdle in characterizing samples [62]: For example, our results suggest that data for some microbiome samples are misclassified as “Homo sapiens” data rather than “human metagenome” data, which makes them much more difficult to locate. As a result, some of the countries listed here with zero samples do have microbiome studies that were either submitted to databases that are challenging to access in bulk (e.g., Zenodo) or mislabeled in the SRA. However, the number of these misclassified samples is likely to be minor, and given the magnitude of differences observed in our study, this is unlikely to affect our main results (see **Materials and methods**). It is also possible that not all samples identified as human in this study are indeed from humans and could, for example, include studies using human gut microbiota transferred into mice. We also did not evaluate differences in host phenotypic information: Most samples are missing even basic information such as sex (77% missing) and age (79% missing), and the most prevalent tag indicating host health status, “host_disease,” is only available for 7.8% of samples (**S1 Table**). Consequently, we do not have sufficient information to draw conclusions about differences in geographic distribution between “healthy” and “disease” samples. Although disease-specific analysis is beyond the scope of our dataset, it would be interesting to investigate differences in the types of microbiome studies, and the questions they ask, on a global scale: If the human microbiome is generally understudied in a given country, it is likely that diseases prevalent in that country may also be lacking information about microbiome associations. We have also limited our database search to 3 databases (SRA, DNA Data Bank of Japan, and European Nucleotide Archive); it is possible that different patterns of global representation are present in other databases, such as MG-RAST [63] and gcMeta [64], although they are orders of magnitude smaller than the NCBI holdings. In addition, as it has been estimated that 20% of microbiome papers do not have publicly available data [65], our study only examines the subset of microbiome studies that also shared their data in the largest international repositories.

Samples collected from the same host could occur in longitudinal studies or datasets in which biological replicates were submitted as separate BioSamples, a pattern that is difficult to evaluate across multiple studies that may identify subjects differently, if at all. If longitudinal studies happen more frequently in some regions than others, it is possible that the reported proportions of samples between countries could differ from the proportions of human subjects. However, given the differences in sample numbers between countries, this is unlikely to change the main results from our study. Moreover, since we are using sample collection as a proxy for investment in microbiome research in a given country, the identity of the subject may not be as relevant—indeed, it is likely more costly to perform a longitudinal study with subject follow-up than it is to recruit more subjects for a single sample each. Still, if longitudinal sampling is more common in studies in North America and Europe (which seems likely, given the extensive infrastructure and funding required for following patients long term), it is possible that the gap between the “Global North” and the rest of the world in terms of microbiome sampling is smaller than our results suggest, if we were to count subjects rather than samples. However, given the magnitude of the difference between countries in our study, we do not believe repeated sampling from the same individuals in the Global North alone can account for such drastic disparities in sample numbers.

To conclude, we analyzed the geographic origins of almost a half-million samples from the largest genomic repositories in the world. We find evidence that the human microbiome field may be encountering some of the same flaws that arose in human genomics [66,67], in which much of the world is excluded and progress is focused on the priorities of the wealthy. The field would benefit from a more global perspective on investigating the human microbiome’s relationship to health and disease.

## Materials and methods

A list of samples was exported from the NCBI BioSample database (https://www.ncbi.nlm.nih.gov/biosample) using the search string “txid408170[Organism] AND biosample sra[filter] AND “public”[filter],” which requests all samples classified under the “human gut metagenome” category in the NCBI Taxonomy (https://www.ncbi.nlm.nih.gov/Taxonomy/Browser/wwwtax.cgi). The resulting sample IDs and all associated tags were loaded into a PostgreSQL database. We repeated this for all categories described as human metagenomes (**Table 2**). We note that the term “human gut metagenome” does not describe the sequencing technique used to generate the microbiome data, including shotgun metagenomics and amplicon sequencing—specifically, 301,700 samples (72.0%) are associated with sequencing runs that list the library strategy as “AMPLICON.”

We then looked in other NCBI categories nested beneath the “organismal metagenomes” category that were not explicitly labeled “human” but were likely to contain some human samples [68]. We downloaded the metadata for samples classified under any NCBI category that was the “generic” version of a human one we had already collected—the “blood metagenome” category is the generic version of the “human blood metagenome” category, for example (**S5 Table**). We downloaded all sample data for any generic categories that had at least 1,000 samples, then evaluated the metadata to find which samples indicated they were taken from a human host. To do this, we used the value of the “host_taxid” field or, if that was blank, the value of “host,” to create a putative “host” value, and manually flagged any that explicitly indicated the sample was from a human—references to “human” or “Homo sapiens,” for example, or if the host included words such as “patient” or “crew member” and did not indicate another species. We evaluated 4,395 unique “host” values for 173,038 samples and found 501 values assigned to 29,934 samples (17.3%) that indicated the host was a human. These were also included in the analysis. The sample data were collected between April and June 2021; to minimize the effect of collecting some body sites after others, only samples dated prior to 2021 were included here.

We then used the NCBI eUtils API to find “runs” associated with each sample, so we could ensure all the BioSamples were associated with actual sequencing data. In the NCBI system, “runs” are the entities associated with sequencing data. We also used this API to obtain information on publication date, library strategy, and the dates on which samples became publicly available. This resulted in a collection of 444,829 samples across 19 body sites (**Table 2**) after removing several hundred samples that were missing dates or sequencing data.

### Representation proportions

To determine which countries were over- or underrepresented relative to their populations, we obtained the 2020 population estimates for all countries as estimated by the United Nations [23]. We used this to calculate 2 percentages for each country, one for the country’s share of the global population and another for the country’s share of human microbiome samples. We then calculated a representation index: For countries with a higher sample percentage than population percentage, we divided the former by the latter to obtain a number indicating how many times more samples are present than expected. For countries with a lower sample percentage than population percentage, we took the negative reciprocal of this number, indicating (in negative numbers) the number one would have to multiply the sample count by to get the number that would be proportionally representative. The interim result leaves overrepresented countries with positive scores and underrepresented countries with negative scores. After removing the scores for countries with 50 or fewer samples, we scaled the positive scores to fall between 0 and 100 and separately scaled the negative scores to fall between 0 and −100. We then plotted these on the map using the “log 10” transformation to add more variation in the color coding for the countries with middling scores. For the regional calculations (**Fig 1C and 1D**), we used top-level classifications from the same United Nations document. Antarctica is not included in a region, so those samples were added to the “Unknown” category for region-level calculations.

To better understand gaps in what data may be available outside of these large centralized repositories evaluated here, we selected several countries with zero attributed samples and did a literature search to determine whether human microbiome studies had been performed there and, if so, where the data are stored. For example, we could not confirm any samples available from Kazakhstan (population 18.7 million) in central Asia, but a human gut microbiome study from there was published in 2020 [69]; its raw sequencing data (but no phenotypic information) are available on Zenodo, a scientific data repository with many submissions but no way of searching for samples or projects. Another Kazakhstan microbiome study [70] is linked to publicly available sequencing data (BioProject PRJEB17632), but with incorrect metadata: Samples are classified as human sequencing data, rather than metagenomic, an issue addressed directly in the SRA submission instructions [71]. In addition, geolocation metadata was submitted, but listed the country of origin as Germany, the location of the senior author (and presumably the sequencing center), rather than Kazakhstan, and the geographical source of the sample, as requested by NCBI [22], although instructions can differ between repositories [62]. A study in Honduras (population 9.9 million) includes SRA data with accurate geolocation information (BioProject PRJEB31759), but the samples were again classified under “Homo sapiens” rather than “human metagenome” [72].

### Visualization

All figures were made using R and the ggplot2 package [73]. Maps use the Equal Earth projection [74] and the rnaturalearth R package [75].

## Supporting information

S1 Fig Samples per year. The x-axis indicates the year, and the y-axis indicates the number of microbiome samples released in that year. Colors indicate the region of origin for each sample and match the colors used in Fig 1C and 1D. The data and code needed to generate this figure can be found at https://doi.org/10.5281/zenodo.5351179.
(TIF)

S1 Table Samples per tag. Each row represents a single metadata field available for BioSample entries. The “samples” column indicates how many samples have a value for that field.
(CSV)

S2 Table Top 10 countries by body site. Each column holds a list of the 10 countries with the most samples in a single body site. The “unknown” category is omitted here.
(CSV)

S3 Table Samples per body site per country. This contains similar data to S2 Table, except no countries or body sites are omitted. Each column is a single body site. Each row is a country, and each cell represents the number of samples from that country that appeared in that body site.
(CSV)

S4 Table Country-level data. Each row represents a single country or territory as defined by the United Nations. There are 10 columns; see the Supporting information documentation for a description of them.
(CSV)

S5 Table NCBI Taxonomy IDs. Each row represents a single body site. The “human” column indicates the ID used to identify samples explicitly labeled as human (e.g., “human gut metagenome”); the “generic” column indicates the ID used to identify samples not labeled as human (e.g., “gut metagenome”). NCBI, National Center for Biotechnology Information.
(CSV)

## Abbreviations

ACCME: African Collaborative Center for Microbiome and Genomics Research
GWAS: genome-wide association study
NCBI: National Center for Biotechnology Information
PRS: polygenic risk score
SRA: Sequence Read Archive
