# Supplementary figures and images for: Public human microbiome data are dominated by highly developed countries
Source: PLoS Biol. 2022 Feb 15;20(2):e3001536. doi: 10.1371/journal.pbio.3001536 (PMC8846514; doi:10.1371/journal.pbio.3001536)

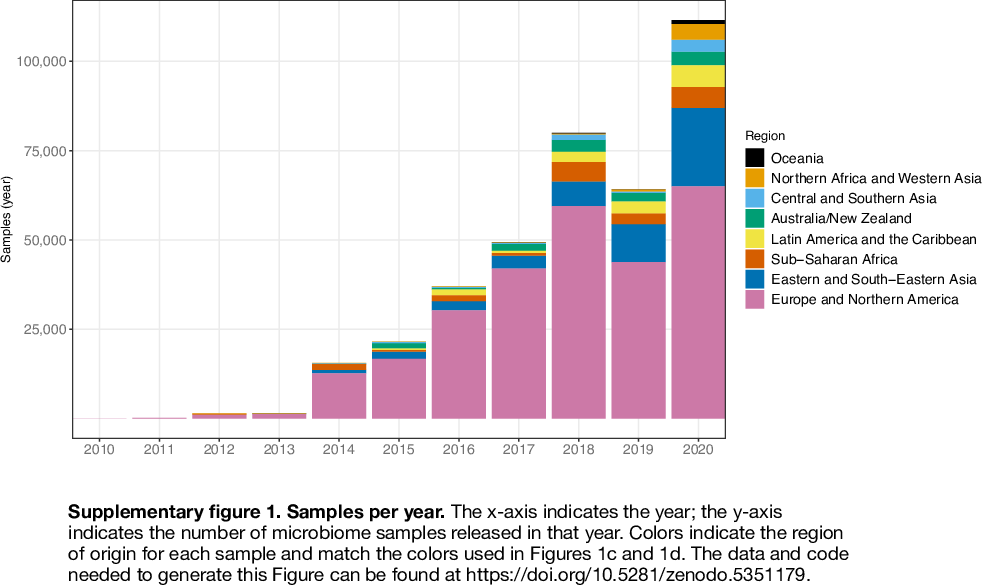

Supplement: S1 Fig — The x-axis indicates the year, and the y-axis indicates the number of microbiome samples released in that year. Colors indicate the region of origin for each sample and match the colors used in Fig 1C and 1D. The data and code needed to generate this figure can be found at https://doi.org/10.5281/zenodo.5351179. (TIF) [file pbio.3001536.s001.tif]
